# A hydrogen sulphide-releasing non-steroidal anti-inflammatory, ATB-346, significantly attenuates human myometrial contractions

**DOI:** 10.1007/s43440-024-00643-z

**Published:** 2024-09-04

**Authors:** Ana Mijušković, Susan Wray, Sarah Arrowsmith

**Affiliations:** 1 Harris-Wellbeing Research Centre, Institute of Life Course and Medical Sciences, University of Liverpool, Liverpool, UK; 2 Department of Life Sciences, Faculty of Science and Engineering, Manchester Metropolitan University, John Dalton Building, Chester Street, Manchester, M1 5GD UK; 3 Department of Physiology, Institute for Biological Research ‘Siniša Stanković’, University of Belgrade, Belgrade, Serbia

**Keywords:** ATB-346, Hydrogen sulphide, Myometrium, Contraction, Tocolytics, Preterm birth

## Abstract

**Background:**

Spontaneous preterm birth is the leading cause of perinatal morbidity and mortality. Tocolytics are drugs used to inhibit uterine contractions in cases of imminent preterm birth, however, few are effective in stopping labour once initiated and all have side effects. Combination approaches involving drugs that target multiple signalling pathways that regulate contractions may increase efficacy, reduce dosage and improve tolerability. Both non-steroidal anti-inflammatory drugs (NSAIDs) and hydrogen sulphide (H_2_S)-releasing compounds can reduce myometrial contractions. In a novel approach we evaluated the tocolytic properties of ATB-346–a H_2_S-releasing derivative of the NSAID naproxen, shown clinically to reduce pain and inflammation in arthritis.

**Methods:**

Using organ baths, paired strips of human myometrium were exposed to increasing concentrations of ATB-346, or equimolar concentrations (10µM and 30µM) of the parent drug, naproxen, or the H_2_S-releasing moiety, 4-hydroxy-thiobenzamide (TBZ), alone. The ability of ATB-346 versus the individual components of ATB-346 to decrease ex vivo spontaneous contractions was investigated, and the potency was compared to a known H_2_S donor, Na_2_S.

**Results:**

Acute application of Na_2_S produced a concentration-dependent decrease in force amplitude and force integral (area under the curve) of contraction. ATB-346 produced a more profound decrease in contraction compared to equimolar concentrations of naproxen or TZB alone and was more potent than the equivalent concentration of Na_2_S.

**Conclusions:**

ATB-346 exhibits potent tocolytic properties in human myometrium. These exciting results call for further exploration of ATB-346, with a view to repurposing this or similar drugs as novel therapies for delaying preterm labour.

## Introduction

Hydrogen sulphide (H_2_S) is an endogenous gaseous signalling molecule, akin to nitric oxide and carbon monoxide. Since the discovery of endogenous H_2_S in the mammalian brain, it has been shown to play a prominent role in many physiological and pathological processes, including blood vessel relaxation, inflammation and cellular protection [1]. In addition to its role in vasodilation, H_2_S has also been found to regulate the contraction of other smooth muscles, including the uterus (myometrium) [2].

In rodent and human myometrium, H_2_S and H_2_S-releasing compounds such the classical H_2_S-releasing donors, sodium hydrosulphide (NaHS) and sodium sulphide (Na_2_S), and the synthetic H_2_S-releasing GYY4137 (morpholin-4-ium 4 methoxyphenyl(morpholino) phosphinodithioate), are potent inhibitors of myometrial contractions [3–6]. H_2_S modulates the activity of several ion channels; K_ATP_, L-type Ca channels [6] and chloride channels [5] in myometrium to dampen excitability. These effects maybe via protein-S-sulphydration affecting their permeability, (as shown for K_ATP_ channels in other tissues [7, 8]) as well as downregulating the expression of proteins involved in contraction [9].

In mammalian tissues, H_2_S is predominantly produced from L-cysteine via the enzymatic activity of cystathionine γ-lyase (CSE), cystathionine β-synthase (CBS) and 3-mercaptosulfurtransferase [10]. Both CSE and CBS are expressed in reproductive tissues during pregnancy including the placenta, fetal membranes and myometrium [3, 11]. Application of L-cysteine also reduces myometrial contraction [12], suggesting that uterine cells can endogenously produce H_2_S. In non-pregnant mouse myometrium, estrogen levels have been shown to regulate CSE and CBS enzyme expression during the estrous cycle [13]. In humans, the expression of these enzymes in myometrium is downregulated towards term and labour onset, [3, 9] and enzyme expression and H_2_S production is also reduced in term and preterm labouring chorionic tissues [14]. Hence, endogenous H_2_S may have an important role in maintaining uterine quiescence which is necessary to continue a pregnancy to term. In accordance with this theory, the inhibitory effects of H_2_S on contraction are reversed in normal labour [6] whilst NaHS has been shown to delay inflammation-induced preterm birth in mice [15].

Preterm birth (before 37 weeks gestation) is the major cause of neonatal death and morbidity with many surviving babies facing significant neurodevelopment delay and lifelong disability [16]. Survival rates and adverse outcomes are strongly associated with gestational age at delivery. Current management of threatened preterm labour is focussed on supressing uterine contractions using tocolytics to delay delivery to allow administration of fetal neuroprotectants and steroids to mature the fetal lungs before delivery [17]. Most tocolytics however, are not very effective, have adverse effects and difficult routes of administration. The non-steroidal anti-inflammatory drug (NSAID), indomethacin, which inhibits cyclo-oxygenase (COX)-mediated production of prostaglandins, is one class of tocolytic. In pre-clinical studies, it is relatively effective in attenuating uterine contractions [18, 19] and clinically, it has been shown to be more effective delaying delivery than other tocolytic agents [20]. Like most tocolytics, however, it is not utero-specific and has maternal and fetal side effects which limits its use to just a few days [21]. There is therefore a pressing need to develop safer and more potent treatments for preterm labour. Combination tocolytics which target different intracellular signalling pathways, are attractive, as they may produce additive inhibitory effects, but allow for lower therapeutic doses to be used, increasing their efficacy and reduce side effects. To date, most combination approaches have focussed on the administration of two drugs, both of which act to suppress cellular pathways which bring about contraction e.g. calcium channel inactivation and blocking the oxytocin receptor [22]. Few have examined approaches involving co-administration of drugs which act to suppress contraction activating pathways as well as activate endogenous pathways which promote relaxation.

A relatively new class of H_2_S donors are the H_2_S-releasing non-steroidal anti-inflammatory drugs (H_2_S-NSAIDs). They consist of a traditional NSAID to which an H_2_S-releasing moiety is covalently attached [23]. ATB-346 (2-(6-methoxynapthalen-2-yl)-propionic acid 4-thiocarbamoyl phenyl ester) is a NSAID derived from naproxen and coupled to 4-hydroxy-thiobenzamide (TBZ), a potent inducer of H_2_S release [24]. Hence, ATB-346, like indomethacin, inhibits COX activity but also releases H_2_S.

Given the potential for ATB-346 as a ‘combined tocolytic,’ stemming from having both anti-inflammatory actions and H_2_S-releasing properties, we examined the tocolytic properties of ATB-346 on ex vivo human myometrial contractions and compared it to the parent NSAID (naproxen), TBZ alone and the H_2_S donor, Na_2_S, which has not previously been investigated in human myometrium.

## Materials and methods

### Reagents

Unless stated otherwise, all reagents were purchased from Merck, UK. ATB-346, and TBZ were kind gifts from Prof John Wallace, University of Calgary, Canada. ATB-346, TBZ and naproxen were prepared in DMSO. Na_2_S, was prepared as described below. All working concentrations were prepared freshly on the day and in the case of Na_2_S, prepared immediately before application to the tissue baths and stored on ice.

### Preparation of Na_2_S

Sodium sulphide (Na_2_S) stock solution was prepared, and concentration determined by a spectrophotometric assay using Ellman's reagent (5,5′-dithiobis-(2-nitrobenzoic acid) or DTNB) which quantifies the concentration of thiol groups in the sample [25]. Briefly, one crystal of Na_2_S was washed twice in molecular grade ultrapure water before dissolving. Serial dilutions were prepared, DTNB added to give 100nM and absorbance read at 412nm. The concentration of Na_2_S was determined using the extinction coefficient of 14,150 M^−1^ cm^−1^ [26]. Stock concentrations of Na_2_S were stored at – 70 °C for up to one week. DTNB was prepared in 0.1M sodium phosphate buffer, 1mM EDTA (pH 8.0) and stored at − 20 °C.

### Sample collection and preparation

Myometrial tissue was obtained with written informed consent from 11 women who underwent elective (pre-labour) caesarean section (CS) delivery. The women were between 23 and 43 years of age, with a singleton pregnancy and delivered between 38 and 41 weeks of gestation. Indications for CS were CS delivery in previous delivery (n = 8), previous traumatic vaginal delivery (n = 2) and breech presentation (n = 1). Women with multiple (e.g. twin) pregnancy, a history of diabetes or hypertension or receiving medications at the time which may affect uterine contractions were excluded from the study. The study received approval from the Local Research Ethics committee (Liverpool East, REC Ref 10/H1002/49 + 5) and institutional review boards of the Research and Development Department, Liverpool Women’s Hospital and University of Liverpool.

During surgery, myometrial tissue was excised from the upper lip of the lower uterine incision site following delivery of the baby and placenta and placed into cooled Hank’s balanced salt solution. Samples were used immediately or within 16 h of collection with storage at 4 °C. In the laboratory, multiple strips (5 × 2 × 1 mm) were dissected along the direction of longitudinal fibres, as previously described [27]. Aluminium clips were attached to each end and the strips were mounted horizontally within 1-mL organ baths continually superfused with physiological saline solution (PSS; in mM: 154 NaCl, 5.6 KCl, 1.2 MgSO_4_, 7.8 glucose, 10.9 HEPES, and 2.0 CaCl_2_) at a rate of 1.0 mL/min, pH 7.4 at 37 °C. One end was attached to a fixed hook, and the other was attached to a FORT 10 g force transducer (World Precision Instruments, Hertfordshire, UK). Each sample was stretched to produce 2 mN force and left to equilibrate in PSS until spontaneous contractions were established, typically within 2 h [28].

### Human myometrial contractility assay

After the onset of spontaneous contractions, strips were left to contract in PSS until a minimum of 4 consecutive contractions of approximately equal amplitude and regular frequency were achieved. The PSS superfusing the baths was then exchanged for PSS containing the relevant compound. Firstly, the effect of applying increasing concentrations of Na_2_S along a log-scale concentration range from 0.3 to 100 µM was investigated (n = 5). Each concentration of Na_2_S was applied for 25 min under continual flow without washout between applications.

In a second experiment involving multiple strips from the same biopsy (paired experiments), the effect of ATB-346 (10 µM and 30 µM, which was guided by the effective concentrations observed for Na_2_S) was compared to the effect of the equivalent concentration of unconjugated naproxen or TBZ alone (n = 5–6). Application of each concentration was for 25 min under continual flow and without washout between applications. The concentration of vehicle (DMSO) in the tissue bath did not exceed 1/1000 dilution and is known not to cause significant changes to contraction [19].

### Data collection and analysis

Contractions were recorded at a sampling rate of 10Hz via a data acquisition system running the associated software (Labscribe 3; World Precision Instruments, UK). Measurement of contractile activity was performed by calculation of the integral area under the tension curve (AUC, arbitrary units) and mean maximum amplitude of contraction (expressed in mN) using Origin Pro 19 (OriginLab Corporation, MA, USA). Control contractile activity was measured in the 25 min preceding the addition of the first drug. The effects of each compound at each concentration were similarly calculated and expressed as a percentage of the integral and amplitude during the control period (i.e., control activity is equal to 100%).

### Statistical analysis

Unless stated otherwise, all values represent the mean ± standard error of the mean (SEM), where “n” is the number of samples, and each represents a different woman. Data were found to be normally distributed by Kolmogorov–Smirnov test, and groups were compared by one-way ANOVA followed by Tukey’s multiple comparison post hoc tests using GraphPad Prism 5.0. A probability value of < 0.05 was taken as level of significance. F values for ANOVA indicate the ratio of the between group variance to within group variance. Values in subscript indicate degrees of freedom for explained variance (number of groups minus 1) and unexplained variance (number of observations minus number of groups/residual variance) respectively.

## Results

### Na_2_S is a potent relaxant of human myometrial contractions.

**Fig. 1:**
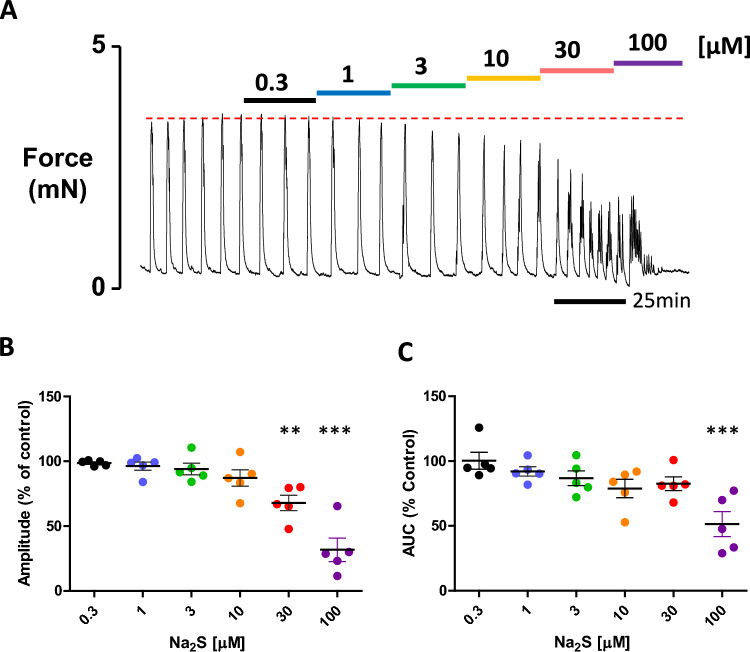
The effect of Na_2_S on human myometrial contractions. **A** Representative recording of spontaneous contractions of human myometrium and the effect of the application of increasing concentrations of Na_2_S (µM). Dotted red line indicates control, pre-treatment activity. **B** and **C** Individual data points, each representing a different woman, showing the effect of Na_2_S on force amplitude and mean integral force (area under the curve, AUC.) respectively. Black bar indicates mean percentage of control activity (± SEM). Data were compared by one-way ANOVA and Tukey’s multiple comparison post-hoc test. P value of < 0.05 was taken as significant, **p < 0.01, ***p < 0.001, n = 5 women

Acute application of the H_2_S donor, Na_2_S, to contracting strips of human myometrium produced a concentration-dependent decrease in contractile activity which was measured based on a decrease in force amplitude and integral of force (area under the curve, AUC) compared to pretreatment control. A representative recording of the effects of Na_2_S is shown in (Fig. 1A). The decrease in force amplitude was determined to be significant at 30µM Na_2_S, causing contraction amplitude to decrease to 67.9% (± 5.87) of control activity with further reduction to 31.8% (± 9.02) of control following application of 100 µM Na_2_S (F_6, 28_ = 23.0, p < 0.0001, Fig. 1B). For integral of force, significance was achieved at 100 µM Na_2_S in which the mean area under the curve was reduced to 51.5% (± 9.62) of control (F_6, 28_ = 7.62, p < 0.0001, Fig. 1C).

### ATB-346 significantly attenuates human myometrial contraction

**Fig. 2:**
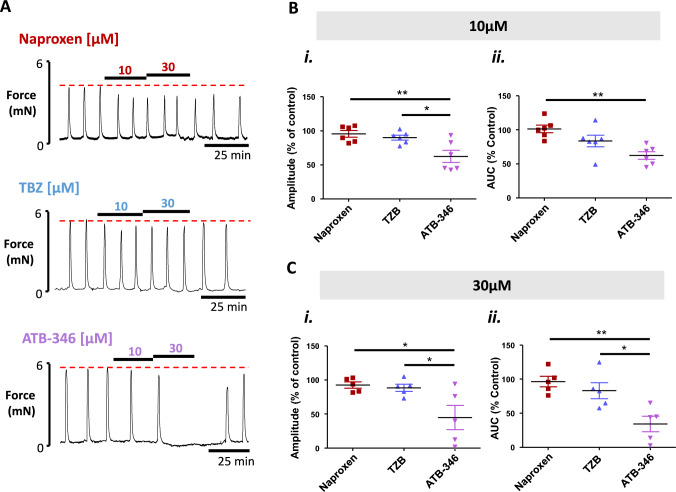
The effect of naproxen, TBZ and ATB-346 on human myometrial contractions. **A** Representative recordings of spontaneous contractions of human myometrium and the effect of the application of naproxen (top trace), TBZ (middle trace) and ATB-346 (bottom trace) in strips from the same woman. Dotted red line indicates control, pre-treatment activity. **B** and **C** The effect of 10µM and 30µM treatment on (i) force amplitude and (ii) mean integral force (area under the curve, AUC) respectively. Mean percentage of control activity is shown (black bars) ± SEM (coloured bars). Data were compared by one-way ANOVA and Tukey’s multiple comparison post-hoc test. P value of < 0.05 was taken as significant where *p < 0.05, **p < 0.01, n = 5–6 women

Application of naproxen or TBZ alone to spontaneously contracting strips of human myometrium (Fig. 2A) resulted in small and non-significant effects on contraction amplitude and AUC. This was true for both concentrations tested: Expressed as percentage of control amplitude and AUC respectively; 10µM naproxen: 95.6% (± 4.8) and 101.4% (± 5.6); 30µM naproxen: 93.6% (± 4.4) and 96.3% (± 7.8); 10µM TBZ: 90.0% (± 3.6) and 83.7% (± 8.5); 30µM TBZ: 88.6% (± 5.2) and 83.0% (± 11.7) (For 10µM amplitude, F_2, 12_ = 8.25, p > 0.05 and AUC, F_2,12_ = 8.57, p > 0.05; For 30 µM amplitude F_2, 14_ = 5.77, p > 0.05 and AUC, F_2, 14_ = 9.88, p > 0.05, Fig. 2B and C).

In contrast however, application of ATB-346 to multiple strips from the same woman produced a significant decrease in activity which was observed at 10µM: 62.4% (± 8.9) of control force amplitude (F_2, 12_ = 8.25, p = 0.0038) and 62.3% (± 5.6) of control AUC (F_2, 12_ = 8.57, p = 0.0033, Fig. 2A, B*i* and *ii*). This effect was further potentiated at 30µM; 44.9% (± 17.8) of control amplitude (F_2, 14_ = 5.77, p = 0.0176) and 34.1% (± 11.4) of control AUC (F_2, 14_ = 9.88, p = 0.0029, Fig. 2Ci and ii). The effect appeared reversible upon washout with contractions typically being restored to their pre-treatment amplitude and force integral within 30 min of washout (observation only).

## Discussion

We present data on human myometrium which suggests that a combination of activating a smooth muscle relaxatory pathway, H_2_S, and inhibiting a contraction promoting pathway (COX), produces significantly more attenuation of uterine contractions, than either action alone. Furthermore, these potent effects were produced using a single drug, ATB-346, not previously tested on uterine smooth muscle. The advantages of this novel approach for the treatment of threatened preterm delivery are discussed.

We found sodium sulphide (Na_2_S) exerts a concentration-dependent tocolytic effect in pregnant human myometrium. This adds to earlier observations on the effect of Na_2_S in non-pregnant rat myometrium [5]. These data also build on findings obtained from other H_2_S donors in rodent and human myometrium, that H_2_S has a relaxant effect on ex vivo myometrial contractions [3–6, 29].

Whilst not directly compared, a greater concentration of Na_2_S was required to achieve a significant decrease in contraction amplitude and AUC than with the hydrogen sulphide-releasing NSAID, ATB-346; the significant effects of ATB-346 were observed at 10µM compared to 30µM (amplitude) and 100 µM (AUC) with Na_2_S. ATB-346 was also more potent than the equivalent concentration of the parent NSAID naproxen, or H_2_S donor, TBZ when applied in isolation. We suggest that the more profound effect of ATB-346 compared to either naproxen or TBZ alone, or Na_2_S, is due to the combination effects resulting from COX inhibition (arising from naproxen moiety) and release of H_2_S via the TBZ moiety, which decreases excitability and thus, contraction [6].

Unlike our data with Na_2_S, the H_2_S-releasing TBZ produced only a small relaxant effect. This can be explained by the previously reported small release of H_2_S from TBZ alone, compared to when it is covalently bound to another drug such as a NSAID like naproxen [30]. In rat liver homogenates the release of H_2_S from ATB-346 was shown to be six times greater than from an equimolar concentration of TBZ [24, 31]. Hence the release of H_2_S from TBZ in our system is unlikely to have been as great as from the equivalent concentration of ATB-346.

Like other NSAIDs, naproxen is known to inhibit prostaglandin synthesis via inhibition of COX-2. In myometrium, early work in pregnant rats showed that a 3-day infusion of naproxen delivered by an implanted osmotic pump, significantly reduced release of prostaglandins from uteri, prolonged gestation and reduced in vivo contractions in response to oxytocin [32]. In non-pregnant humans, naproxen therapy has also been shown to reduce menstrual prostaglandin levels and severity of menstrual cramps (dysmenorrhea) compared to placebo [33]. Here we show that acute application of naproxen to ex vivo pregnant human myometrium reduces contractions, but not significantly under the concentration range we tested. This appears to be the first paper examining the effect of acute application of naproxen ex vivo in human myometrium. Interestingly, the acute application of another NSAID, indomethacin, to ex vivo strips of myometrium causes significant decreases in contractions—~ 60% at 30 µM [19]. Higher concentrations of naproxen may be required to achieve the equivalent tocolytic effects as indomethacin. However, these concentrations are likely to exceed the normal therapeutic range.

Before the onset of term and preterm labour, the expression of pro-inflammatory cytokines (including chemokines) in uterine tissues is increased [34]. These inflammatory mediators stimulate the expression of contraction-associated proteins including oxytocin receptors, connexin 43, and prostaglandin H synthase in myometrium and production of prostaglandins which ultimately leads to labour onset [35, 36]. H_2_S donors have also been shown to affect the expression of proinflammatory cytokines and contraction-associated protein expression in human myometrial cells by interfering with NFκB signalling and inhibiting the production of the proinflammatory cytokines IL-1β, IL-6 and TNF-α [9, 37], as well as to delay lipopolysaccharide-induced preterm labour in mice [15].

ATB-346 was originally developed to reduce gastrointestinal ulceration and injury caused by NSAIDs such as naproxen, when taken for chronic pain such as in osteoarthritis. Multiple pre-clinical studies have since demonstrated a superior anti-inflammatory effect of ATB-346 when compared to naproxen whilst also having less damaging effects on the gastrointestinal tract [24, 38, 39]. More recently in a human model of bacterial infection in skin, ATB-346 was shown to reduce neutrophil infiltration [40], and in mice, ATB-346 provides neuroprotection in traumatic brain injury models by reducing secondary inflammation and tissue injury[41]. It would therefore be interesting to examine whether ATB-346 has similar anti-inflammatory activities in the myometrium. If so, the anti-inflammatory properties of H_2_S acting in concert with the anti-inflammatory effects associated with inhibition of COX-2 via naproxen, combined with its direct tocolytic actions would present a promising novel therapeutic strategy for reducing rates of preterm birth. For example, in addition to its tocolytic properties in slowing contractions in women in threatened preterm labour, it could potentially be used prophylactically to prevent upregulation of inflammatory signalling pathways and prevent preterm labour in women at high risk of preterm birth.

A limitation to our investigation was that we were unable to study the effects of ATB-346 in preterm myometrium which would better reflect the tissue type in which tocolytics would be used, or in the presence of hormones such as oxytocin and PGF_2α_, which in vitro can reduce potency of NaHS and GYY4137 in myometrium [6]. This action is not particular to H_2_S, as it has been found for other tocolytics [19, 22, 42]. Thus, future studies should investigate whether the potency of ATB-346 is reduced in the presence of these hormones and test its potency in tissues from preterm births. The mechanism by which contractions are reduced by ATB-346 also requires investigation. For example, some NSAIDs, including naproxen, are also known to effect cAMP signalling [43], whilst others, e.g. indomethcin, affect calcium influx [18]. Hence, there may be additional actions to those traditional concepts of inhibition of COX-2 and prostaglandin synthesis.

## Conclusion

There is an urgent need for better tocolytic (and prophylactic) treatments to prevent or delay preterm birth. Combination approaches targeting different contraction pathways enable greater potency whilst also reducing therapeutic dosages and thereby decreasing maternal/fetal side effects. As with other smooth muscles, H_2_S exerts potent relaxatory effects in pregnant human myometrium. The novel and exciting feature about H_2_S-releasing NSAIDs in the myometrium is their dual action; supressing contraction (and inflammatory) activation pathways (via COX inhibition) whilst also activating endogenous relaxatory pathways (via H_2_S). H_2_S-releasing NSAIDs may therefore be a promising alternative therapy for prevention of preterm labour and should be further explored.

## Data Availability

The datasets generated and analysed during the current study are available from the corresponding author upon reasonable request.
